# Methods for qPCR gene expression profiling applied to 1440 lymphoblastoid single cells

**DOI:** 10.1016/j.ymeth.2012.10.004

**Published:** 2013-01

**Authors:** Kenneth J. Livak, Quin F. Wills, Alex J. Tipping, Krishnalekha Datta, Rowena Mittal, Andrew J. Goldson, Darren W. Sexton, Chris C. Holmes

**Affiliations:** a Fluidigm Corporation, 7000 Shoreline Court, Suite 100, South San Francisco, CA 94080, USA; b Department of Statistics, University of Oxford, Oxford OX1 3TG, United Kingdom; c Stem Cell Laboratory, UCL Cancer Institute, University College London, London WC1E 6BT, United Kingdom; d UEA Flow Cytometry Services, BioMedical Research Centre, School of Biological Sciences, University of East Anglia, Norwich NR4 7TJ, United Kingdom; e BioMedical Research Centre, Norwich Medical School, University of East Anglia, Norwich NR4 7TJ, United Kingdom

**Keywords:** Single-cell gene expression profiling, High throughput qPCR, Real-time PCR, Microfluidic arrays, Eukaryotic transcription, Stochastic noise in gene expression

## Abstract

The stochastic nature of generating eukaryotic transcripts challenges conventional methods for obtaining and analyzing single-cell gene expression data. In order to address the inherent noise, detailed methods are described on how to collect data on multiple genes in a large number of single cells using microfluidic arrays. As part of a study exploring the effect of genotype on Wnt pathway activation, data were collected for 96 qPCR assays on 1440 lymphoblastoid cells. The description of methods includes preliminary data processing steps. The methods used in the collection and analysis of single-cell qPCR data are contrasted with those used in conventional qPCR.

## Highlights

► Microfluidic arrays enable analysis of 96 qPCR assays on 1440 single cells. ► Detailed methods on obtaining qPCR data and performing preliminary data processing. ► Data from sufficient cells to address noise inherent in single-cell transcription. ► Methods used for conventional qPCR do not necessarily apply to single-cell qPCR.

## 1 Introduction

Single-cell analysis has been called “the new frontier in Omics” [1]. For a variety of reasons and using a variety of techniques, researchers are analyzing cellular heterogeneity by collecting genomics data at single-cell resolution [2]. Relying on average measurements will often be misleading when the cells being studied are heterogeneous. By applying single-cell techniques, the role of cell heterogeneity in complex phenomena such as stem cell differentiation and cancer development can now be directly assessed.

In the study of single-cell gene expression, one of the most provocative findings is that eukaryotic transcription occurs in pulses. This is shown most directly by the results of Chubb et al. [3]. They detected nascent transcripts of *dscA*, the *discoidin Ia* gene, directly in living *Dictyostelium* cells. For this gene, they measured a mean burst duration of 5.2 min and a mean interval of inactivity of 5.8 min, but there was a great deal of stochastic variation in each of these parameters. It is important to note that they were able to detect nuclear transcripts because of the high intensity caused by having multiple nascent chains at the gene locus. Background fluorescence prevented detection of individual RNA molecules in the cytoplasm. Thus, the pulsing observed by Chubb et al. represents the behavior of the transcriptional machinery, not the accumulation and overall level of mRNA molecules per cell.

Raj et al. [4] used *in situ* hybridization to count individual mRNA molecules in fixed Chinese hamster ovary (CHO) cells and thus determine the overall level of transcripts per cell. Like Chubb et al., they observed transcriptionally active and inactive nuclei, albeit statically rather than dynamically. Because they could detect cytoplasmic transcripts as well, Raj et al. observed that these transcriptional pulses, or bursts, lead to massive variation in the total number of mRNA molecules per cell. There were a few cells with a relatively high number of transcripts; whereas, most cells had a much more modest number of transcripts. Furthermore, cells with transcriptionally active nuclei tended to have a much higher number of mRNA molecules per cell than cells with inactive nuclei. Raj et al. conclude that eukaryotic transcripts are produced in short but intense bursts interspersed with intervals of inactivity during which transcript levels decay. Up- or downregulation of transcription can be accomplished by changing either burst size or burst frequency.

Bengtsson et al. [5] used qPCR to quantify transcripts for five genes in a total of 169 individual cells isolated from mouse pancreatic islets. Their study had the advantage over previous biochemical measurements of mRNA in single cells in that they examined a sufficient number of cells in order to meaningfully assess the distribution of transcript levels among a population of single cells. Their basic conclusion was that, for each gene, the number of transcripts detected per cell exhibit an approximate lognormal distribution. This is, in fact, the same sort of skewed distribution reported by Raj et al. namely, a few cells with a relatively large number of transcripts and most cells with a much smaller number. Fig. 1 in Bengtsson et al. reports the results for *ActB* expression levels in 96 cells and it indicates only four cells with over 1000 transcripts per cell and 40 cells with zero to 100 transcripts/cell. Thus, the finding of an approximate lognormal distribution is consistent with the transcriptional pulsing reported by Chubb et al. and Raj et al. Using digital PCR, Warren et al. [6] found a similar skewed distribution of *Gapdh* transcripts in individual mouse hematopoietic progenitor cells.

We embarked on a study to investigate if single-cell gene expression profiling would provide useful insights into the problem of associating genetic variation with cell phenotype. Lymphoblastoid cell lines from 15 genotyped individuals were treated with a Wnt pathway agonist. For each cell line, qPCR was used to obtain single-cell gene expression profiles for 48 baseline cells and 48 perturbed cells. Thus, data were collected from a total of 1440 single cells. The biological findings of this study will be published elsewhere. This paper describes the nuts and bolts of how the data were obtained and the preliminary processing used to prepare the data for higher order statistical analyses. It is important to document these methodological details because the noise inherent in single-cell gene expression data, presumably due to transcriptional pulsing, challenges conventional methods for obtaining and analyzing qPCR data. Factors such as replicates, data display, limit of detection, and normalization need to be re-evaluated.

## 2 Material and methods

### 2.1 Cells

#### 2.1.1 Culture conditions

The following cell lines were obtained from Coriell Institute (Camden, NJ, USA): GM10838, GM10839, GM10860, GM10861, GM07029, GM07019, GM12239, GM12801, GM12802, GM12864, GM12865, GM12752, GM12753, GM07048, GM06991, and GM11881. All samples were seeded at 4 × 10^5^ cells/mL in standard media (RPMI 1640 containing l-glutamine [Life Technologies; 21875], 15% Fetal Calf Serum [GE Healthcare; A15–104], and Penicillin/Streptomycin [100 Units mL^−1^/100 mg mL^−1^ final concentration; Life Technologies; 15140-122]). In order to avoid batch to batch variations for cell growth, the standard media for all cell cultures were obtained from single batches of each of the cell culture constituents. Cells were initially passaged in T-25 flasks with all perturbations occurring in 24-well plates. Passage numbers were the same for all cells lines used and never exceeded six. Treatment with 22.5 mg/mL acycloguanosine (Acyclovir) to suppress EBV activity was not found to have any observable effect on growth or gene expression and was, thus, omitted. Seeded cells were grown for an initial 24 h, then perturbed with 10 μM SB216763 [7] or left unperturbed (baseline) for a further 24 h, before sorting.

#### 2.1.2 Single cell sorting

A BD FACS Aria II (Becton Dickinson) flow cytometer was used to perform single cell sorting following the manufacturer’s aseptic sort protocol. Cells were counted and viability accessed using a hemocytometer and trypan blue dye exclusion prior to staining. Nuclear DNA was stained using Hoechst 33342 (2 μg/mL) in buffer (pH 7.2) containing HBSS, 20 mM HEPES (Invitrogen), 5.55 mM glucose, 10% Fetal Calf Serum, 50 μM Verapamil for 90 min at 37 °C, with gentle vortexing every 15 min. Cells were subsequently stained with PE-Cy7 CD27 (eBioscience) and Biotin IgM (BD Biosciences) antibodies for 20 min and Streptavidin APC-eFluor 780 (eBioscience) secondary antibody staining for a further 15 min. Antibody concentrations used were those recommended by the manufacturer and all antibody staining was performed on ice in the Hoechst buffer specified above. Hoechst 33342 staining was detected using 375 nm laser illumination and 450/40 nm band pass filtered detection; PE-Cy7 CD27 was detected using 488 nm laser excitation and 780/60 nm band pass filtered detection; and IgM APC-eFluor 780 was detected using 633 nm laser excitation and 780/60 nm band pass filtered detection. Individual cells were sorted using the following gating criteria: debris discrimination using forward and orthogonal 488 nm laser scatter (cells selected), doublet discrimination using orthogonal pulse height and width (individual cells selected), nuclear DNA content (G_0_/G_1_ selected), IgM expression (IgM**^−^** selected) and CD27 expression (CD27**^−^** selected). In order to obtain maximum purity, cells were sorted twice using the defined gating strategy. Initially, sorted cells were collected as a pooled sample and subsequently re-sorted for single cell deposition directly into pre-aliquoted Lysis Solution (see Section 2.4 cDNA synthesis).

### 2.2 Single-cell qPCR assays

DELTAgene assays (Fluidigm) were designed for 96 human transcripts. The genes and primer sequences are given in Table 1. Ribosomal RNA was deliberately not included as a target because it was feared that the extremely high abundance of ribosomal RNA would saturate the preamplification process, which was performed for 20 cycles in order to obtain sensitivity down to a single cDNA molecule (see Section 3.1.4). Whenever possible, assays are designed to cross an intron. Even when assays do not cross introns, the number of genomic copies of any amplicon is typically only two, so the presence of genomic DNA is generally not a concern for single-cell analysis of transcript levels. The predicted melting temperatures of the primers and the amplicon lengths are similar to those in TaqMan gene expression assays and thus the primers are expected to behave similarly in preamplification. The oligos were synthesized by IDT and dissolved at a concentration of 200 μM in buffer consisting of 10 mM Tris–HCl, pH 8.0; 1 mM EDTA. First, for each assay, a Primer Pair Mix was prepared containing 50 μM Forward Primer and 50 μM Reverse Primer by mixing 20 μL 200 μM Forward Primer, 20 μL 200 μM Reverse Primer, and 40 μL buffer consisting of 10 mM Tris–HCl, pH 8.0; 0.1 mM EDTA; 0.25% Tween-20 (Thermo Scientific PI-28320). In order to prepare 10 × Preamplification Primer Mix (500 nM each primer), 10 μL of each of the 96 Primer Pair Mixes (50 μM each primer) was mixed with 40 μL buffer consisting of 10 mM Tris–HCl, pH 8.0; 0.1 mM EDTA; 0.25% Tween-20. In order to prepare 10× Assay (5 μM each primer) each Primer Pair Mix was diluted by mixing 10 μL Primer Pair Mix (50 μM each primer) with 90 μL buffer consisting of 10 mM Tris–HCl, pH 8.0; 0.1 mM EDTA; 0.25% Tween-20.

### 2.3 Testing of assays with cDNA prepared from bulk RNA

**Fig. 1:**
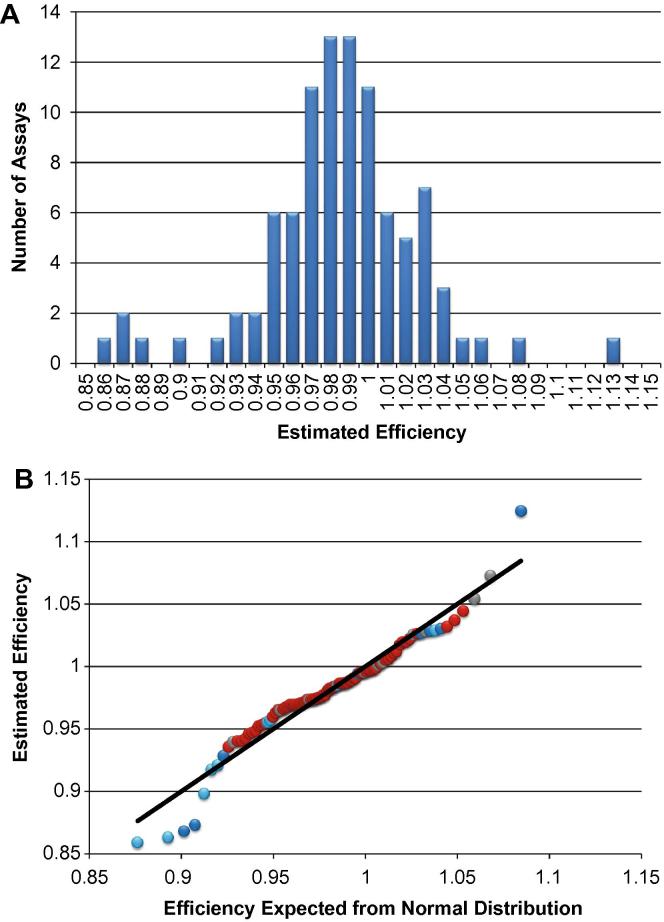
Distribution of estimated efficiencies for 95 qPCR assays detecting human transcripts. Panel A is a histogram displaying the efficiencies estimated from the slopes of standard curve plots. The average efficiency of this distribution is 0.98 with a standard deviation of 0.042. Panel B is a Q–Q plot with the experimental estimated efficiencies plotted on the *y*-axis and the values expected for a normal distribution with mean efficiency = 0.98 and standard deviation = 0.042 plotted on the *x*-axis. The black line indicates the values expected for a normal distribution (*y* = *x*). For the 85 efficiency values determined using GM12802 RNA, the data points are depicted as light blue (derived from plots with 3 points in the standard curve), dark blue (4 points in the standard curve), or red (⩾5 points in the standard curve). The 10 efficiency values determined using Universal Human cDNA are depicted in gray and all of these values are derived from standard curves with at least 5 points. It can be seen that the points that deviate the most from a normal distribution are all derived from standard curves with only 3 or 4 points. Such determinations are probably more prone to error than those derived from standard curves with 5 or more points.

The assays were tested with Universal Human cDNA (BioChain C4234565-R) and with cDNA prepared from bulk total RNA extracted from cell lines GM06991, GM10839, GM12801, and GM12802. This was done in order to confirm that each assay had the expected quantitative response with dilution of template and to determine the expected *T*_m_ for the specific amplicon for each assay. As an example, an experiment performed using GM12802 cDNA will be described. Preamplification was performed in a 20-μL reaction containing cDNA prepared from approximately 20 ng GM12802 total RNA, 50 nM each Preamplification Primer, and 1× Applied Biosystems TaqMan® PreAmp Master Mix (4391128). The thermal cycling protocol was: 95 °C, 10 min; 14 cycles of (96 °C, 5 s; 60 °C, 4 min); 4 °C hold. Unincorporated primers were digested by adding an 8-μL solution containing 40 units Exonuclease I (New England BioLabs M0293L) in 1× Exonuclease I Reaction Buffer and using the thermal protocol: 37 °C, 30 min; 80 °C, 15 min; 4 °C hold. Reactions were diluted by adding 72 μL buffer consisting of 10 mM Tris–HCl, pH 8.0; 1 mM EDTA to each sample. Fourteen 1:2 dilutions were prepared by mixing 30 μL cDNA sample with 60 μL buffer consisting of 10 mM Tris–HCl, pH 8.0; 1 mM EDTA; 0.25% Tween-20. These dilutions were made in 1.5-mL tubes with vortexing and centrifugation after each dilution. The 15 cDNA samples (spanning over 6 orders of magnitude) and 1 No Template Control (NTC, 10 mM Tris–HCl, pH 8.0; 1 mM EDTA; 0.25% Tween-20) were analyzed by qPCR using 96.96 Dynamic Array™ IFCs and the BioMark™ HD System from Fluidigm as described below in Section 2.6 with the following modification. Twenty microliters of the Supermix/Loading Reagent mix were dispensed to each of 16 wells in a 96-well assay plate, then mixed with 15 μL cDNA or NTC sample. Each of these samples was dispensed 6 times into Sample Inlets of the 96.96 IFC so there were 6 technical qPCR replicates for each sample. In order to minimize reduction in precision due to sampling error (see Section 3.1.5), only sample/assay combinations where specific amplification was detected for all replicates were used in preparing standard curves of Log_10_ Sample Dilution versus average *C*_q_ value. For each assay, efficiency was estimated from the slope of the standard curve using the formula Efficiency = [10^(−1/slope)] minus 1. The experiment was performed twice (two 96.96 arrays) so the slope for each assay was determined two times. Eleven of the assays had less than three points in their standard curves. For 10 of these assays, an efficiency estimate was available from a similar experiment performed using the Universal Human cDNA and these values were used. For one assay (for gene *HNF4A*), an efficiency estimate was not determined. Fig. 1A shows the distribution of estimated efficiencies for 95 of the 96 assays used in this study. Fig. 1B shows a quantile–quantile (Q–Q) plot demonstrating that most of the efficiency values are close to the values expected for a normal distribution with mean efficiency = 0.980 and standard deviation = 0.042. The implication of finding a normal distribution is that the variation observed reflects mainly the errors involved in estimating efficiency. The distribution in Fig. 1A is consistent with the hypothesis that at least 90 assays have efficiencies of approximately 98% and up to 5 assays have efficiencies in the range 87–90%.

### 2.4 cDNA synthesis

Thermo-Fast® 96 PCR Plate Non-Skirted 96-well PCR plates (Thermo Scientific AB-0600) were used for the collection of single cells. Single cells were collected directly into 5 μL Lysis Solution consisting of 10 mM Tris–HCl, pH 8.0; 0.1 mM EDTA; 0.5% NP40 (Thermo Scientific PI-28324); 0.1 units/μL SUPERase In™ (Ambion AM2696). Lysed cells were frozen on dry ice, then stored at −80 °C. In order to synthesize cDNA, the plate of lysed cells was thawed on ice, centrifuged at 1500 rpm for 1 min, and transferred to a thermal cycler (Applied Biosystems® GeneAmp® PCR System 9700) already at 65 °C. After 90 s incubation at 65 °C, the plate was transferred to ice while the thermal cycler was still at 65 °C. After incubating on ice for at least 1 min, the plate was centrifuged and 1 μL qScript™ cDNA SuperMix (Quanta Biosciences 95048-100) was added to each well. Following a brief vortex and centrifugation, the plate was transferred to a thermal cycler and subjected to the following thermal protocol: 25 °C, 5 min; 42 °C, 30 min; 85 °C, 5 min; 4 °C, hold. At this point, the plate can be stored at −20 °C.

### 2.5 Preamplification

Preamplification was performed on 96 cDNA samples prepared as above in 96-well PCR plates (USA Scientific 1402-9700). A mix was prepared containing 800 μL 2× TaqMan® PreAmp Master Mix plus 160 μL 10× Preamplification Primer Mix (500 nM each primer), and 9 μL of this mix was added to each cDNA sample. Following a brief vortex and centrifugation, the plate was transferred to a thermal cycler and subjected to the following thermal protocol: 95 °C, 10 min; 20 cycles of (96 °C, 5 s; 60 °C, 6 min); 4 °C hold. Reactions were then treated with Exonuclease I in order to digest the primers. A mix was prepared containing 128 μL 20 units/μL Exonuclease I, 64 μL 10× Exonuclease I Reaction Buffer, plus 448 μL H_2_O, and 6 μL of this mix was added to each sample. Following a brief vortex and centrifugation, the plate was transferred to a thermal cycler and subjected to the following thermal protocol: 37 °C, 30 min; 80 °C, 15 min; 4 °C hold. Reactions were diluted by adding 54 μL buffer consisting of 10 mM Tris–HCl, pH 8.0; 0.1 mM EDTA to each sample. Following a brief vortex and centrifugation, samples were stored at −20 °C.

### 2.6 Single-cell qPCR

Preamplified cDNA samples from single cells were analyzed by qPCR using 96.96 Dynamic Array™ IFCs and the BioMark™ HD System from Fluidigm. Processing of the IFCs and operation of the instruments were performed according to the manufacturer’s procedures. Each experiment consisted of analyzing 96 samples with 96 DELTAgene assays. In order to prepare samples for loading into the IFC, a mix was prepared consisting of 420 μL Sso Fast EvaGreen Supermix with Low ROX (BioRad 172-5212), 42 μL 20× DNA Binding Dye Sample Loading Reagent (Fluidigm 100-3738), plus 18 μL H_2_O, and 4 μL of this mix was dispensed to each well of a 96-well assay plate (Axygen Scientific, P-96-450-V-C). Three microliters of preamplified cDNA sample was added to each well and the plate was briefly vortexed and centrifuged. Following priming of the IFC in the IFC Controller HX, 5 μL of the cDNA sample + reagent mix were dispensed to each Sample Inlet of the 96.96 IFC. For the DELTAgene assays, 4.5 μL of each 10× Assay (5 μM each primer) were dispensed to each Detector Inlet of the 96.96 IFC. After loading the assays and samples into the IFC in the IFC Controller HX, the IFC was transferred to the BioMark HD and PCR was performed using the thermal protocol GE Fast 96 × 96 PCR + Melt v2.pcl. This protocol consists of a Thermal Mix of 70 °C, 40 min; 60 °C, 30 s, Hot Start at 95 °C, 1 min, PCR Cycle of 30 cycles of (96 °C, 5 s; 60 °C, 20 s), and Melting using a ramp from 60 °C to 95 °C at 1 °C/3 s. Data was analyzed using Fluidigm Real-Time PCR Analysis software using the Linear (Derivative) Baseline Correction Method and the Auto (Global) Ct Threshold Method. The *C*_q_ values determined were exported to Excel for further processing.

### 2.7 Digital PCR

**Fig. 2:**
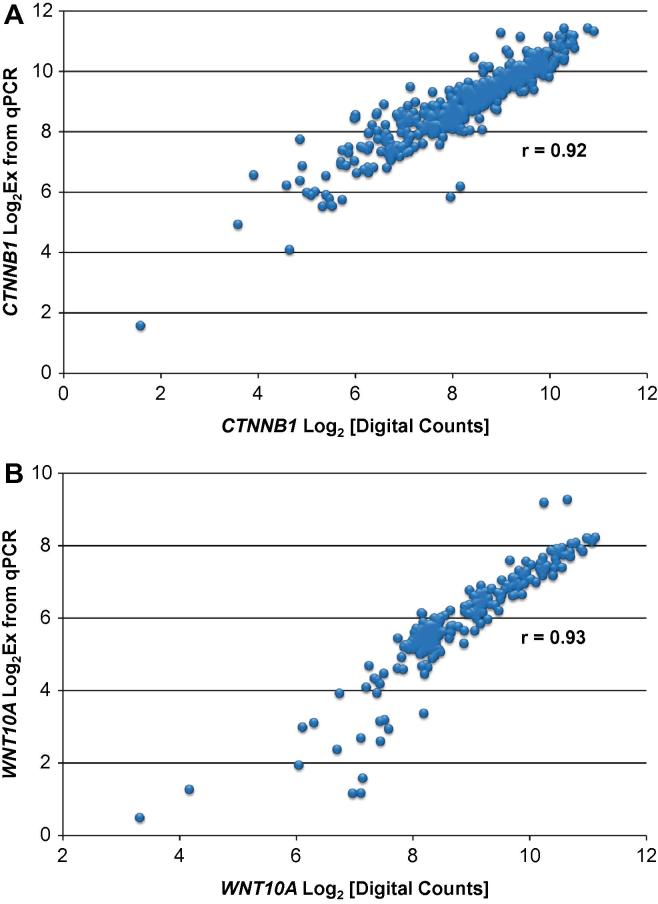
Correlation of qPCR and digital PCR single-cell results for transcripts from two genes. Plots show qPCR results plotted on the *y*-axis and digital PCR results plotted on the *x*-axis for *CTNNB1* (A) and *WNT10A* (B). The results are for individual cells from 9 of the cell lines (GM07029, GM07019, GM12239, GM12864, GM12865, GM12752, GM12753, GM06991, GM11881). The qPCR values are Log_2_Ex values determined as described in Section 2.8.3. The digital PCR values are the log base 2 values of the number of target molecules estimated to be present in each panel of the 48.770 IFCs.

For two of the assays, the preamplified cDNA samples from single cells of 9 cell lines were analyzed by digital PCR using 48.770 Dynamic Array IFCs (Fluidigm) and the BioMark HD System. For analysis with the *WNT10A* assay, the preamplified cDNA samples were diluted 1:8 in buffer consisting of 10 mM Tris–HCl, pH 8.0; 0.1 mM EDTA; 0.25% Tween-20. For analysis with the *CTNNB1* assay, the preamplified cDNA samples were diluted 1:64 in buffer consisting of 10 mM Tris–HCl, pH 8.0; 0.1 mM EDTA; 0.25% Tween-20. In order to prepare samples for loading into the IFC, a mix was prepared consisting of 200 μL Sso Fast EvaGreen Supermix with Low ROX, 40 μL 20× DNA Binding Dye Sample Loading Reagent, plus 40 μL 10× Assay (5 μM each primer), and 5 μL of this mix was dispensed to each of 48 wells in a 96-well assay plate. An aliquot (2.1 μL) of diluted preamplified cDNA sample was added to each well and the plate was briefly vortexed and centrifuged. Following priming of the IFC in the IFC Controller MX, 5 μL of the cDNA sample + reagent mix were dispensed to each Sample Inlet of the 48.770 IFC and 10 μL H_2_O was dispensed to each of the sixteen Hydration Inlets. After loading the reactions into the IFC in the IFC Controller MX, the IFC was transferred to the BioMark HD and PCR was performed using the thermal protocol: Hot Start at 95 °C, 1 min, PCR Cycles of 2 cycles of (96 °C, 5 s; 66 °C, 40 s) and 30 cycles of (96 °C, 5 s; 64 °C, 20 s). Data was analyzed using Fluidigm Digital PCR Analysis software using the Linear (Derivative) Baseline Correction Method, the User (Global) Ct Threshold Method with threshold set at 0.01, and a Ct Range of 12 to 28 cycles. The software determines the number of positive PCR reactions for each of the 48 panels and then uses a Poisson correction to estimate the number of target molecules present in each panel. Fig. 2 shows the correlation between qPCR and digital PCR results for the assays for *CTNNB1* (2A) and *WNT10A* (2B). Quantification with digital PCR depends critically upon the assumption that a single target molecule will generate a positive amplification plot. Thus, the good correlation between qPCR and digital PCR results indicates that if a single target molecule is present in a PCR reaction chamber, it will almost always be detected.

### 2.8 Data processing

#### 2.8.1 Culling cells with low expression levels

For a population of cells being treated as homogeneous, determine the fraction of cells (*p_i_*) that are positive for each assay. Thus, *p_i_* represents the probability of detection success for the *i*^th^ assay. Then, assign a failure index (*f_i_*) for each reaction by setting *f_i_* = 1 for detection failure and *f_i_* = 0 for detection success. The Detection Failure Score is determined for each cell by summing *f_i_* × *p_i_* across all assays for that particular cell. A cell is culled from the data set if its Detection Failure Score is greater than 3× the median Detection Failure Score for the population.

#### 2.8.2 Estimating standard deviation due to sampling error

The standard deviation of *C*_q_ values due to sampling error was estimated using a population size of 1000 reactions. For a given average number of molecules per reaction volume, the Poisson distribution was used to determine the number of reactions containing 0, 1, 2, etc. molecules. For reactions with one or more molecules, a population was established containing Log_2_(number of molecules) values. For example, at an average concentration of one molecule per reaction volume, the population contained 368 Log_2_(1) values, 184 Log_2_(2)values, 61 Log_2_(3) values, 15 Log_2_(4) values, 3 Log_2_(5) values, and 1 Log_2_(6) value. For each average number of molecules per reaction volume, the standard deviation of this population is an estimate of the contribution of sampling error to variation when measuring *C*_q_ values.

#### 2.8.3 Applying limit-of-detection (LOD) *C*_q_

C_q_ values were converted to expression levels using the equation Log_2_Ex = LOD (Limit of Detection) *C*_q_–*C*_q_ [Assay]. If this value is negative, then the result is assigned ND for not detected. Log_2_Ex represents transcript level above background expressed in log base 2. In order to decide on a reasonable value for LOD *C*_q_, different values were tried starting with 30 and reducing in one cycle increments. At each LOD *C*_q_ value, the Fano factor (variance [*σ*^2^] divided by mean [*μ*]), *F*, was calculated for each assay across the population of cells being considered. LOD *C*_q_ = 24 was the lowest integral value at which the Fano factor for all assays was greater than or equal to one. Thus, LOD *C*_q_ = 24 was used because it is expected the data should at least exhibit Poisson noise (*F* = 1). At lower LOD *C*_q_ values, the elimination of positive detection data is reducing variation for some of the assays below this Poisson noise threshold. Log_2_Ex values are converted to a linear scale by calculating 2^(Log_2_Ex). This value is referred to as the idealized number of transcripts above background because it assumes a qPCR efficiency of one. Although actual numbers may be somewhat lower, the shapes of the distributions do not change drastically for the range of efficiencies shown in Fig. 1A. For multivariate analysis or other purposes, ND can be replaced with zero so that all data points have a numerical value.

#### 2.8.4 Cell-to-cell median normalization

Ignoring the ND values, the median Log_2_Ex value is determined for each cell. These values are averaged across all the cells being considered. For each cell, an offset value is determined by taking the average median value and subtracting the median value for that particular cell. Normalization is accomplished by adding this offset to all Log_2_Ex values for that cell. ND results are still designated as ND. This shifts the Log_2_Ex distribution for each cell so that all cells in the population being considered have the same median Log_2_Ex value. The validity of using a median Log_2_Ex value based on only 96 genes was investigated by running a simulation using data from a preliminary experiment on biological replicates of cell line GM10860. In order to prepare the bioreplicates, the process of seeding, growth for 48 h with no perturbation, and sorting to collect 96 single cells was performed two times, with sorting occurring on different days. The two batches of single cells were analyzed using the 96 qPCR assays. In order to run the simulation, the following process was repeated 10,000 times: (1) Randomly select n assays from both of the bioreplicates (*n* = 1–96); (2) Median normalize the results for the n assays in both bioreplicates; (3) Record the Pearson correlation of the Q–Q plot per assay; (4) Take the median correlation of all assays. A plot (Fig. 7) was prepared of n, the number of assays used to normalize, versus the median correlation. The better the two bioreplicates compare, the closer the correlation will be to 1.

## 3 Results and discussion

### 3.1 Detection failure

#### 3.1.1 Level of detection failure

Data were collected for 15 cell lines and, for each cell line, there were two experimental conditions, baseline and perturbed. Thus, there were a total of 30 distinct sample types. For each sample type, 48 single cells were analyzed for a total 1440 single cells. As data were collected for 96 assays, there are a total of 138,240 qPCR data points. The simplest analysis of these data is plus/minus detection, that is, was the assay target detected in the single cell or not. Of all the data points, 53.0% (73,307 data points) were positive for specific target amplification and 47.0% (64,933 data points) were negative. The expectation is that many, if not most, of the detection failures indicate the transcript was not present in the cell. One of the consequences of transcriptional pulsing is that most cells have a relatively low number of transcripts for any particular gene. For example, in Raj et al. [4], the top histogram in Fig. 6B indicates that the number of transcripts encoding the large subunit of RNA polymerase II is zero to four in 74 cells and greater than four transcripts in only 29 cells. This is for a transcript that encodes a protein that will be present in every cell. Still, there are technical reasons for detection failure, so these will be considered one at a time. As detailed below, the only major technical contributor to detection failure should be variation in the reverse transcriptase reaction. This effects plus/minus detection only at low transcript levels. Thus, in these experiments, detection failure predominantly indicates no or only very few transcripts present in the cell.

#### 3.1.2 Lysis

**Fig. 3:**
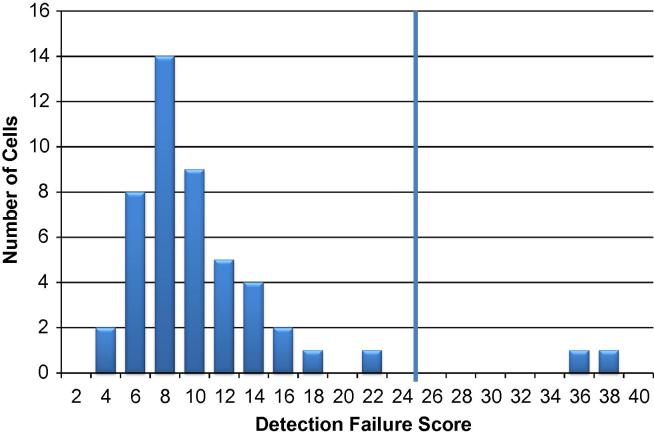
Distribution of Detection Failure Scores for one group of 48 cells. Detection Failure Scores were calculated for the 48 GM11881 baseline cells as described in Section 2.8.1. The vertical line at Detection Failure Score = 24 is the 3× median threshold used to cull low expressing cells from the data set.

Incomplete lysis should produce a global drop in the transcripts detected per cell. Thus, it is useful to have a metric that assesses if the overall level of transcripts is unusually low in a particular cell. We have devised a scoring system based on counting the number of assays not detected in each cell. The contribution of each assay, though, is weighted based on the success rate of that assay in the population being considered. Thus, failing to detect an assay that is detected in 90% of cells receives a score of 0.9 and has a larger effect on the overall score than failing to detect an assay that is detected in only 10% of cells and thus has a score of 0.1. In this study, scoring was performed separately for each of the 30 sample types. Fig. 3 shows the distribution of scores for one set of 48 cells. A cutoff of greater than 3 × median was used to eliminate cells from further analysis. In the example shown in Fig. 1, two cells had scores above the cutoff and were culled from the data set. Overall, 20 cells (1.4%) were eliminated from further analysis. This process removes cells that are compromised not just due to incomplete lysis, but for any reason that lowers overall transcript levels, e.g., imprecise sorting or apoptosis. One attribute of this culling method is that it is based on data from all the gene targets in the study, obviating the need to include pre-selected control genes expected to be detected in every cell.

#### 3.1.3 Reverse transcriptase reaction

The reverse transcriptase step is the main contributor to technical variation in reverse transcription-qPCR quantification [8] and thus is probably the biggest technical cause of detection failure in our data. For plus/minus detection, the most critical parameter is reverse transcriptase efficiency. The effect of reverse transcriptase efficiency is difficult to easily characterize because it depends on the gene, the location of the target amplicon within the transcript, and the exact protocol used. In their single cell study, Bengtsson et al. [9] report efficiencies ranging from 8% to 99% for assays detecting five different gene transcripts. It appears, though, that reverse transcriptase efficiency remains consistent from sample to sample as long as the same assay and same exact protocol are used [8]. Thus, reverse transcriptase efficiency is the major factor that determines absolute detection limit in qPCR analysis of single-cell gene expression. We did not assess reverse transcriptase efficiency for the assays and protocol that we used. So, it should be expected that the limit of detection in terms of transcripts per cell varies from assay to assay. The protocol was designed, though, so that, if one cDNA molecule is generated, there is a high probability that that molecule will be detected (see Sections 3.1.4 and 2.7).

#### 3.1.4 Preamplification

**Fig. 4:**
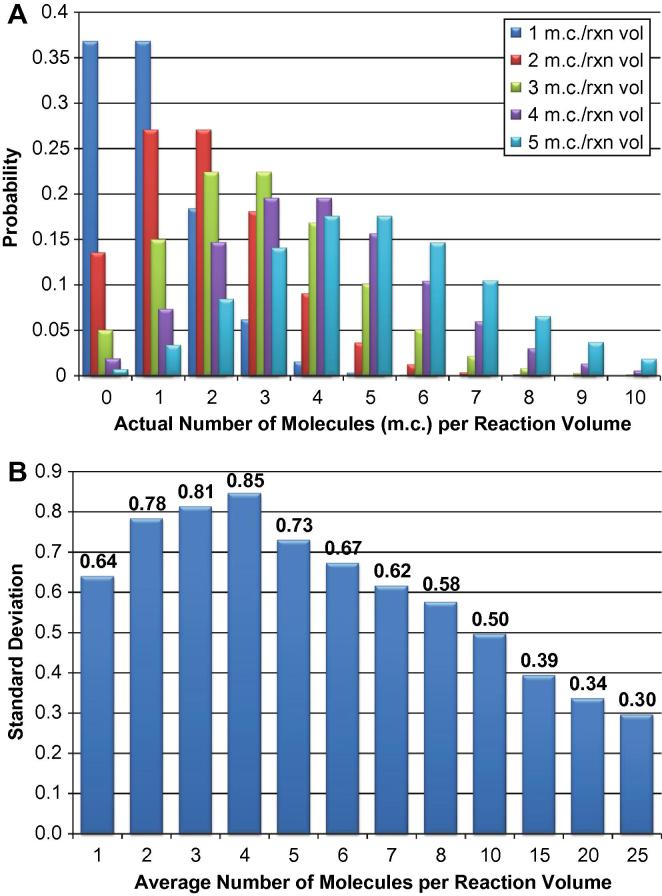
Effect of sampling error on qPCR results. Panel A depicts Poisson distributions at low concentrations of target molecules per qPCR reaction volume, ranging from 1 to 5 molecules per reaction volume. Panel B shows the calculated effect (see Section 2.8.2) of the Poisson distribution on *C*_q_ standard deviation values at various low concentrations of target molecules per qPCR reaction volume.

Multiplex preamplification enables the detection of multiple targets from a single cell. In these experiments, preamplification was performed for 20 cycles in order to increase the concentration of all 96 targets so that each would be robustly detected when the sample was distributed across 96 reaction chambers. What is robust detection? Poisson statistics indicates that at an average concentration of 5 targets per reaction volume, there is a 99.3% chance that any reaction will contain at least one molecule (see Fig. 4A). Thus, ideally, preamplification should amplify one cDNA molecule to a concentration that corresponds to five molecules per reaction volume. In the 96.96 Dynamic Array IFC, the volume of the sample chamber for each reaction is 6.85 nL. Thus, 5 targets per reaction volume correspond to 730 molecules/μL. Table 2 shows how many cycles of preamplification are required to amplify one single-stranded cDNA molecule to a concentration of at least 730 molecules/μL as a function of preamplification efficiency. Thus, if preamplification efficiency is at least 90%, then 20 cycles of preamplification should ensure a greater than 99% probability of detecting one original cDNA molecule with one replicate. Applied Biosystems does not provide a “specification” for the PCR efficiency of its TaqMan® PreAmp Master Mix. In the protocol for this master mix (P/N 4384557), they do state on p.21: “Typically, 90% of targets produce ΔΔ*C*_T_ values within ± 1.5.” Table 3 shows the expected ΔΔ*C*_q_ (or ΔΔ*C*_T_) values after 14 cycles of preamplification (as prescribed in the manual) if the only source of variation is preamplification efficiency. The fact that the ±1.5 value must include sources of variation other than PCR efficiency means it is likely that TaqMan PreAmp Master Mix achieves at least 90% efficiency for 90% of assays. Furthermore, the validation results using standard RNAs reported by Devonshire et al. [10] show that preamplification can have efficiencies close to 100%.

#### 3.1.5 Sampling error

Fig. 4A shows that as target concentration falls below 5 target molecules per reaction volume, the probability of having no target in any given reaction increases. Thus, some detection failures may be due to this sampling effect. This is more likely for assays with a preamplification efficiency less than 90%. Also, stochastic effects during the first or second round of preamplification may delay attainment of the optimal target concentration. The overall contribution of sampling error to detection failure, though, should be relatively small because of the design of the preamplification step. Replicates could be run to reduce the contribution of sampling error to detection failure, but this comes with a significant increase in experimental cost. Although the effect on detection failure should be minor, sampling error still has an effect on quantification precision at low transcript levels. Fig. 4B shows the contribution of sampling error to the standard deviation of *C*_q_ values at low concentrations of target molecules per reaction volume. In our data, this effect starts to increase variation at a *C*_q_ value of approximately 18.

#### 3.1.6 Non-specific amplification

Although use of a DNA binding dye (in this case, EvaGreen) in qPCR detects non-specific amplification, it also enables identification of non-specific amplification through *T*_m_ analysis. Out of the 64,933 detection failures, 3,265 (5.0%) were reactions that failed because the *T*_m_ of the amplification product was lower than the *T*_m_ expected for the specific target amplicon. Thus, non-specific amplification was only a minor contributor to the overall number of detection failures. Non-specific amplification was prevalent in only ten of the 96 assays, so the contribution of non-specific amplification could have been drastically reduced by replacing these ten assays with primer pairs that generated minimal non-specific amplification.

### 3.2 Transcript distributions

#### 3.2.1 Display of data

**Fig. 5:**
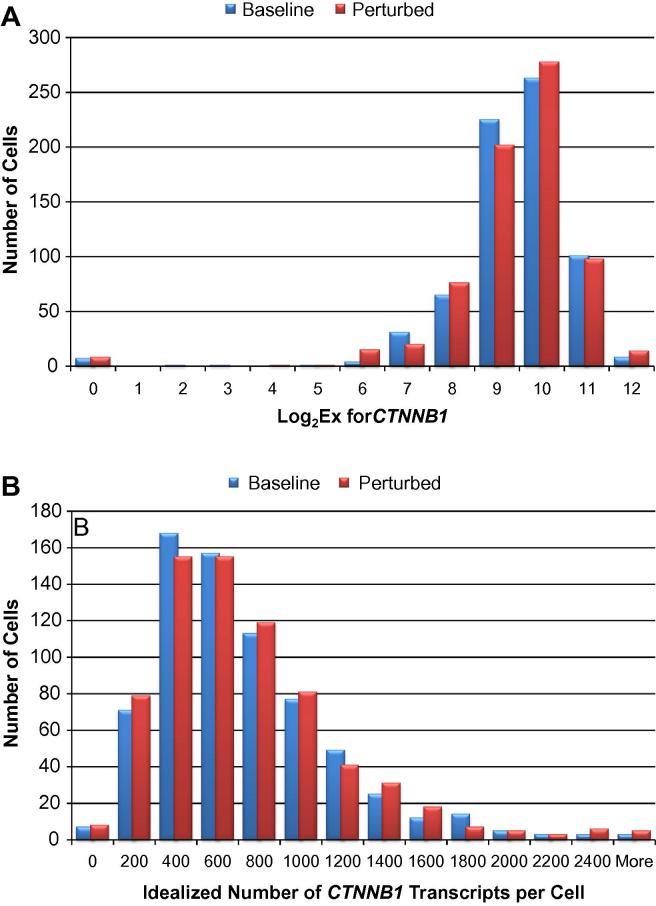
Histograms showing *CTNNB1* transcript levels in lymphoblastoid cells in logarithmic (A) and linear (B) scale. Conversion of *C*_q_ values to Log_2_Ex values and conversion from logarithmic to linear scale are described in Section 2.8.3. For these distributions, the data for baseline (blue) and perturbed (red) cells for all 15 cell lines were pooled. The zero bins show the number of cells in which *CTNNB1* transcript was not detected.

Because of the variation inherent in single-cell gene expression, it is important to assess the population behavior of each transcript. This can be done by using histograms that bin expression levels and display the number of cells in each bin. For transcripts in our study, we observed the skewed distribution reported by others. Namely, there are a few cells with a relatively high number of transcripts and most cells have a much lower number of transcripts. Fig. 5 shows the distributions of *CTNNB1* transcripts in baseline and perturbed cells on both log (5A) and linear (5B) scales. These and the other distributions we observe could be described as lognormal. A more thorough analysis of these distributions will be reported in the paper describing the effect of genotype on expression in these single cells.

#### 3.2.2 Replicates

**Fig. 6:**
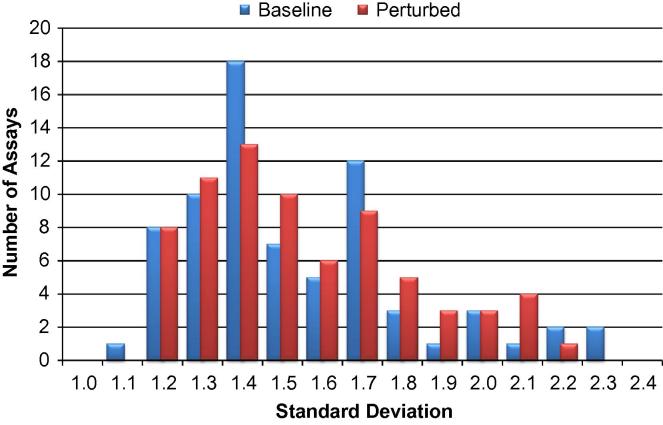
Distributions of standard deviations for the 73 assays expressed in at least 10% of the cells. Results are shown separately for pooled baseline (blue) and perturbed (red) cells.

Because it was expected that biological variation would be much greater than qPCR technical variation, only single qPCR replicates were performed for each cell. This assumption was confirmed by the data in Fig. 6, showing the distributions of standard deviations for assays detected in at least 10% of the cells. The total variation observed (median standard deviation of approximately 1.4 cycles) is much greater than the maximum technical variation typically observed in qPCR experiments (0.15–0.25 cycles). By running single replicates, it was possible to collect data on a large number of single cells and keep the cost of the study within reason.

#### 3.2.3 Normalization

**Fig. 7:**
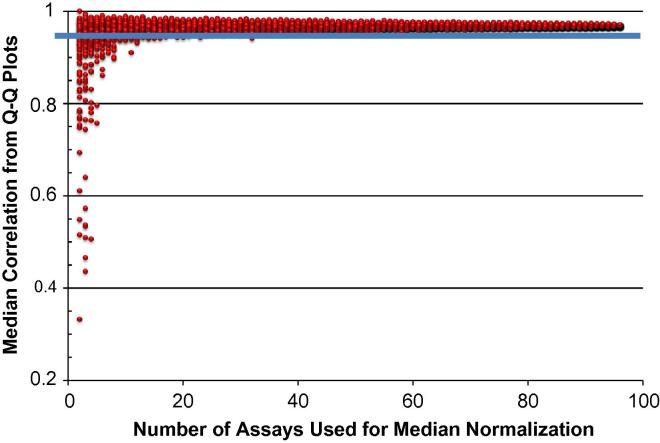
Results of a simulation testing the robustness of using data from 96 assays to estimate the median expression level per cell. The details of the simulation are described in Section 2.8.4. The *x*-axis plots the number of randomly selected assays (out of 96 assays) that were used to determine a median Log_2_Ex value for each cell. These median Log_2_Ex values were used to normalize the results for two batches of 96 single cells. The *y*-axis is a measure of how well the results from the two batches of cells correlate after normalization. The blue horizontal line is at a Pearson correlation value of 0.95.

In order to avoid any unintended bias, we decided not to use cell-to-cell normalization in our subsequent analyses. Basically, the data are already normalized on a per cell basis. If additional cross-sample normalization is performed, the use of a single reference gene is precluded because of the large cell-to-cell variation of any one gene. Some researchers have used the average of two or more reference gene to normalize their data [11]. The geNorm method described by Vandesompele et al. [12] is a robust way to derive a normalization factor from multiple reference genes. Section 2.8.4 describes a normalization method that shifts Log_2_Ex values so that all cells in a given population have the same median Log_2_Ex value. One advantage of this method is that it is based on data from all detected genes, not just the results from a few pre-selected reference genes. One danger of this method is that it is attempting to assess the overall transcript level in a cell on the basis of data from only 96 genes. Fig. 7 shows the results of the simulation described in Section 2.8.4 designed to explore the robustness of using a limited number of assays to estimate the median transcript level per cell. The largest number of assays that shows any median correlation below 0.95 is 32 assays. Therefore, the use of 96 assays seems to be justified for estimating a median transcript level. The validity of this or any other normalization method needs to be assessed in the context of the specific experiments being conducted. For our particular set of data, the use of median normalization did not significantly improve the correlation observed for each of the 96 assays in Q-Q plots comparing bioreplicates and thus median normalization was not used.

### 3.3 Concluding remarks

High throughput, cost-effective methods are required in order to collect enough data from a sufficient number of cells to characterize the noise inherent in single-cell gene expression. This paper documents the ability to collect gene expression data for 96 qPCR assays on 1440 individual cells by using microfluidic arrays. Performing such a study using conventional qPCR in plates would be cost prohibitive because of the large volume of qPCR master mix required. Also, the use of microfluidic arrays greatly reduces the time and labor required to collect the data.

In addition to reporting the detailed methods on collecting the data, this paper documents some of the preliminary data processing steps that can be used in analyzing single-cell qPCR data. These basic steps include culling low expressing cells, applying a limit-of-detection value to convert *C*_q_ values to expression values, and displaying population data in expression histograms. Basic steps used in conventional qPCR, such as running multiple replicates and normalizing to reference genes, do not necessarily apply to the collection and analysis of single-cell gene expression data.

## Figures and Tables

**Table 1:** Genes and primer pairs for the assays used in this study.

Gene	Ensembl Gene ID	Forward primer	Reverse primer	Comment
*ACTB*	ENSG00000075624	CGACCAACCGCGAGAAGATGAC	CGTTAGCACAGCCTGGATAGCAA	
*ADAR*	ENSG00000160710	CGAGCACTGTTGACCCACTTCC	CGTCAGATGCCCTTGGCTGAAAA	
*APC*	ENSG00000134982	CGAGCACTTGTGGCCCAACTAAA	CGTCGCCAAGACAAATTCCTCAAAAC	
*AXIN1*	ENSG00000103126	CGACAAGGAGCTGCTGACCAAAA	CGTCACCACCCCACAGTCAAAC	
*AXIN2*	ENSG00000168646	CGAGTCCACGGAAACTGTTGACA	CGTGTGGCTGGTGCAAAGACATA	
*BCL9*	ENSG00000116128	CGAACTCCAGCCAAAGTGGTGTA	CGTCAACCTGGCCCTTCAAAACA	
*BTRC*	ENSG00000166167	CGATAAGCGGCCTTCGAGACAA	CGTAACCTGTATGGCCTGTGAGAA	
*CASP2*	ENSG00000106144	CGAAACTGCCCAAGCCTACAGAA	CGTTTGGTCAACCCCACGATCA	
*CCND1*	ENSG00000110092	CGAAGAGGCGGAGGAGAACAAA	CGTAGGGCGGATTGGAAATGAAC	
*CCND2*	ENSG00000118971	CGAGCAGAAGGACATCCAACCCTA	CGTTCTTCGCACTTCTGTTCCTCA	
*CCND3*	ENSG00000112576	CGACCGACAGGCCTTGGTCAA	CGTTGGCGGGTACATGGCAAA	
*CDH1*	ENSG00000039068	CGAAGTGCCAACTGGACCATTCA	CGTTCTAAGGCCATCTTTGGCTTCA	
*CDH3*	ENSG00000062038	CGAGAAGATGACACCCGTGACAA	CGTTGGAGCTGGGTGATGTCATA	
*CDKN1A*	ENSG00000124762	CGATGGAGACTCTCAGGGTCGAAAA	CGTCGGCGTTTGGAGTGGTAGAA	
*CSNK1G1*	ENSG00000169118	CGATTGACCTCTGTGACCGAACA	CGTGTGCACGTATTCCATTCGAGAA	
*CSNK2A1*	ENSG00000101266	CGATCCGAGTTGCTTCCCGATAC	CGTCAACCCAAACTCCACATATCCAAA	
*CTBP1*	ENSG00000159692	CGAGAGCACAACCACCACCTCA	CGTGGGCTGTGTTCACCAGGAA	
*CTNNB1*	ENSG00000168036	CGAAGCTCTTACACCCACCATCC	CGTTGCATGATTTGCGGGACAAA	
*DAAM1*	ENSG00000100592	CGAAGCCCACAAATGCCCTGAAA	CGTTCGGTCCATACTGTTCCTTCCA	
*DAB2*	ENSG00000153071	CGACCCACCTCCACAAAGTACCA	CGTGATGTCTGATGCAAGCAAGTCA	
*DACT1*	ENSG00000165617	CGAATCTGAAGAGCACCTGGAGAC	CGTGCCCCATCACTCAGCTCATA	
*DKK1*	ENSG00000107984	CGACGGGCGGGAATAAGTACCA	CGTGGACTAGCGCAGTACTCATCA	
*DKK3*	ENSG00000050165	CGAAATGGGACCATCTGTGACAACC	CGTGCAAAGCTCGCCCTCCA	
*DVL2*	ENSG00000004975	CGATGCCTCCCGCCTCCTTAA	CGTTGACGCTGCTGAAGGATGAC	
*EIF4E*	ENSG00000151247	CGAACTTCTTATTGCAAGGCAGTCTCTA	CGTGTGCTCCAAACTTATGCTGTTCA	
*ELAC1*	ENSG00000141642	CGAGGAAAAGAAACGCCCAGGTAA	CGTAGCTTCCCATAGGCAGGAC	
*FGF9*	ENSG00000102678	CGAGGTGTGGACAGTGGTCTCTA	CGTCCCTAAAGATGCATTCGGAAGTA	
*FGF20*	ENSG00000078579	CGACCAGGGAACCAGGAAAGACC	CGTCCTTCTCATTCATCCCGAGGTA	
*FOXN1*	ENSG00000109101	CGAACCTGGATGCCATCAATCCC	CGTGGGCCAAGCTATCATCCTTCA	
*FOXO1*	ENSG00000150907	CGAGGTGTCAGGCTGAGGGTTA	CGTTTCTCTCAGTTCCTGCTGTCA	
*FOXO3*	ENSG00000118689	CGACACTGAGGAAGGGGAAGTGG	CGTGAGAGCAGATTTGGCAAAGGG	
*FRZB*	ENSG00000162998	CGACCTCTGCCCTCCACTTAATGTTA	CGTCAGCTATAGAGCCTTCCACCAA	
*FZD1*	ENSG00000157240	CGAGGCAACCTTGCCTTTGAGAA	CGTCCAGGTGACCTCAACATTTCC	
*FZD2*	ENSG00000180340	CGACTGCGCTTCCACCTTCTTCA	CGTAATGATAGGCCGCTCTGGGTA	
*FZD5*	ENSG00000163251	CGATGGGGACTGTCTGCTCTTCT	CGTTGGGGAGAGACGGTTAGGG	
*FZD8*	ENSG00000177283	CGACGTGGTCTTCTTGCTGGTCTA	CGTAGGAACCATGTGAGCGACAA	
*GADD45A*	ENSG00000116717	CGAGCGACCTGCAGTTTGCAATA	CGTTTTGCTGAGCACTTCCTCCA	
*GAPDH*	ENSG00000111640	CGAACACCATGGGGAAGGTGAAG	CGTGTGACCAGGCGCCCAATA	
*GSK3A*	ENSG00000105723	CGACGCCATCAAGAAGGTTCTCC	CGTTTGCAGTGGTCCAGCTTAC	
*GSK3B*	ENSG00000082701	CGAACTACCAAATGGGCGAGACA	CGTATGGTAGCCAGAGGTGGATTAC	
*GTSE1*	ENSG00000075218	CGAGGGCGATCCCTGTTCCA	CGTTCCTTGCGAGATTGCTGGTA	
*HDAC9*	ENSG00000048052	CGAGGGCCAACTGGAAGTGTTAC	CGTATGCGTTGCTGTGAAACCA	
*HNF4A*	ENSG00000101076	CGAGTGCGGAAGAACCACATGTAC	CGTAGTAGCGGCACTGGTTCC	
*ICT1*	ENSG00000167862	CGAAAAGCAAGCCGACAGTGAC	CGTCAGGACCACTACTCCGACAATA	
*ID2*	ENSG00000115738	CGAAGACCCGGGCAGAACCA	CGTCACACAGTGCTTTGCTGTCA	
*JAG1*	ENSG00000101384	CGAAACAAAGGCTTCACGGGAAC	CGTCAAGTGCCACCGTTTCTACAA	
*JUN*	ENSG00000177606	CGAAAGAACTCGGACCTCCTCAC	CGTTGGATTATCAGGCGCTCCA	
*KREMEN1*	ENSG00000183762	CGAAGAGCACGAGGATGGTGTCTA	CGTTTGTAGCAGCCAAGGTTTCCA	
*LDLR*	ENSG00000130164	CGACACCACGGTGGAGATAGTGAC	CGTTTCTCATTTCCTCTGCCAGCAA	
*LEF1*	ENSG00000138795	CGAAAGAAAGTGCAGCTATCAACCA	CGTGCTGTCTTTCTTTCCGTGCTA	
*LRP5*	ENSG00000162337	CGACTGCGCCTCACACTACAC	CGTGGCAGATTTCTGGCTGAACA	
*LRP6*	ENSG00000070018	CGAGACAGACCTGGACACCAACTTA	CGTGGATGAGGCAAGTCATCTGCTA	
*MAP3K7*	ENSG00000135341	CGACGAATCATGTGGGCTGTTCA	CGTACGAGTCATCAGGCTCTCAA	
*MAPK10*	ENSG00000109339	CGATCATCCTGGGGATGGGCTA	CGTTTTGTGGCGAACCATTTCTCC	
*MET*	ENSG00000105976	CGACAGAGACTTGGCTGCAAGAA	CGTCATGTCTCTGGCAAGACCAAA	
*MINPP1*	ENSG00000107789	CGATCCTCCAGTTTGGTCATGCA	CGTTGTACGCTGTTAGGGGTTCC	
*MMP7*	ENSG00000137673	CGAGTGAGCTACAGTGGGAACA	CGTTCTCCTTGAGTTTGGCTTCTAA	
*MYC*	ENSG00000136997	CGACTCCTTGCAGCTGCTTAGAC	CGTCGAGTCGTAGTCGAGGTCATA	
*NKD1*	ENSG00000140807	CGAGGCTCCAAGAAGCAGCTGAA	CGTTACAGGGTGAAGGTCCACTCC	
*NLK*	ENSG00000087095	CGAAGACATTAAGCCAGGGAATCTCC	CGTCTTCCACTCTGGCCAATCCA	
*NPPC*	ENSG00000163273	CGACCAACGCGCGCAAATACAAA	CGTCAGCTTGAGGCCGAAGCA	
*POLR2A*	ENSG00000181222	CGACTCGCCTCTTCTACTCCAACA	CGTATGGAGTCCCCAATGCCAATA	
*PPARD*	ENSG00000112033	CGAGGCAAAGCCAGCCACAC	CGTGCCATTCACCAACTGCTTCC	
*PPIA*	ENSG00000196262	CGATCTGGTTCCTTCTGCGTGAA	CGTCACCCAGGGAATACGTAACCA	
*PPP2CA*	ENSG00000113575	CGAGTGGTAACCAAGCTGCAATCA	CGTCTACGAGGTGCTGGGTCAA	
*PPP2R1A*	ENSG00000105568	CGAGTTGCCAATGTCCGCTTCAA	CGTTCTAGGATGGGCTTGACTTCAC	
*PPP2R5E*	ENSG00000154001	CGACAACCCAGCATTGCCAAAA	CGTGAGGGTCTTCGCTGTCAAA	
*PRKCA*	ENSG00000154229	CGAACCATCCGCTCCACACTAAA	CGTAGTCGTCGGTCTTTGTCTGAA	
*PRKCE*	ENSG00000171132	CGATATCTTCGGCAGCCCACCTA	CGTGACACTGGTATCCCTGCTTTCC	
*PYGO1*	ENSG00000171016	CGATATCCTGGCTTTGGAGGCTA	CGTACCACAGTATGGGGAAGACA	
*RAC1*	ENSG00000136238	CGACTCCTGTAGTCGCTTTGCCTA	CGTAGAACATCGTCAGCACTAGCA	
*RARS*	ENSG00000113643	CGAAGCTGCTACTGTGTGGAGAA	CGTCAGCATACGCCACATGTTCA	
*SOX17*	ENSG00000164736	CGACACAACGCCGAGTTGAGCAA	CGTGCTCTGCCTCCTCCACGAA	
*T*	ENSG00000164458	CGACGCTTCAAGGAGCTCACCAA	CGTGCCAGACACGTTCACCTTCA	
*TBP*	ENSG00000112592	CGATGCCCGAAACGCCGAATATA	CGTCGTGGTTCGTGGCTCTCTTA	
*TCF4*	ENSG00000196628	CGAAGCCTGCATCCACATGAAC	CGTACATCGGAGGAAGACTGGAA	
*TCF7*	ENSG00000081059	CGATAAGGAGAGCGCTGCCATCA	CGTTTGCGGGCCAGCTCATAGTA	
*TCF7L1*	ENSG00000152284	CGATCTCCCCAGAGATCGATCCA	CGTGAGAGTGGGTAATACGGTGACA	
*TCF7L2*	ENSG00000148737	CGACGCTTTGGCCTTGATCAACA	CGTCCTTCACCTTGTATGTAGCGAAC	Whole gene assay
*TCF7L2*	ENSG00000148737	CGAATCATGATCCCCGACCTGAC	CGTGTGCTGCCGGACTGAAAA	RefSeq NM_001146274.1
*TCF7L2*	ENSG00000148737	CGACCCCTCAGACTTCACTGTCA	CGTGCACCACTGGCACTTTGTTA	RefSeq NM_001146283.1
*TCF7L2*	ENSG00000148737	CGACATGTCTTTGAATTTGGAATATTACAATG	CGTCCTTCACCTTGTATGTAGCGAAC	RefSeq NM_030756.4
*TCF7L2*	ENSG00000148737	CGAAGCTTCATATGCAACTGTACCC	CGTGGCTGCTTGTCCCTTTTCC	RefSeq NM_001198528.1
*TNFRSF11A*	ENSG00000141655	CGACTTCTCTGCCAGCTAGAAAACC	CGTAGACGCGAAGAGAAGCAGAA	
*TOP2B*	ENSG00000077097	CGAGATGCTGCAAGCCCTCGTTA	CGTGGTTGTCATCCACAGCAGGAA	
*USMG5*	ENSG00000173915	CGAACTGGCCACATATGGAAGCA	CGTCAGATGAGGTTAAGAACCGTAGACA	
*VEGFC*	ENSG00000150630	CGAGCCAACCTCAACTCAAGGAC	CGTGCATGCATTGAGTCTTTCTCCA	
*WIF1*	ENSG00000156076	CGACATCTGCCCACCTGGATTCTA	CGTACAGGTCCCTCCATTAAAGCA	
*WNT1*	ENSG00000125084	CGACGCTTCCTCATGAACCTTCAC	CGTCGTGGCACTTGCACTCC	
*WNT10A*	ENSG00000135925	CGAGACTCGCAACAAGATCCCCTA	CGTGCGATGGCGTAGGCAAAA	
*WNT11*	ENSG00000085741	CGAGGCGTGTGCTATGGCATCAA	CGTGCAGTGTTGCGTCTGGTTCA	
*WNT16*	ENSG00000002745	CGACACCACGGGCAAAGAAAACAA	CGTTGGCAGCGGCAGTCTAC	
*WNT2B*	ENSG00000134245	CGACCGGGCCCTCATGAACTTA	CGTACTCACGCCATGGCACTTA	
*WNT3A*	ENSG00000154342	CGAGCCCCACTCGGATACTTCTTA	CGTGAGGAATACTGTGGCCCAAC	
*WNT4*	ENSG00000162552	CGAAGAGCCCTCATGAACCTCCA	CGTCCGTGGCACTTGCATTCC	
*WNT5B*	ENSG00000111186	CGACTTCTGACAGACGCCAACTCC	CGTGCTGGGCACCGATGATAAACA	

**Table 2:** Theoretical number of molecules generated by preamplification from one single-stranded cDNA molecule as a function of cycles and efficiency.

Cycles	Efficiency (%)	Number of molecules generated	Concentration (number of molecules/μL)	
			In 75 μL^a^	After dilution with qPCR reagents
18	100	131,072	1748	749
19	95	166,197	2216	950
20	90	197,842	2638	1131
21	85	220,513	2940	1260

a Volume of sample at the end of the preamplification step.

**Table 3:** Effect of efficiency on ΔΔ*C*_q_ after 14 cycles of preamplification.^a^

Efficiency	100%	95%	90%	85%	80%
ΔΔ*C*_q_	0.0	0.5	1.0	1.6	2.1

a Calculated assuming one assay at 100% and the other at the indicated efficiency.
